# Supplementary material for: Carroll–Schrödinger equation as the ultra-relativistic limit of the tachyon equation
Source: Sci Rep. 2025 Apr 22;15:13884. doi: 10.1038/s41598-024-82010-9 (PMC12015531; doi:10.1038/s41598-024-82010-9)
Supplement: Supplementary file 1 — Supplementary Information. [file 41598_2024_82010_MOESM1_ESM.pdf]

# Supplementary material for:

# "Carroll-Schrödinger Equation"

Mojtaba Najafizadeh[1,2]

[1] *Department of Physics, Faculty of Science, Ferdowsi University of Mashhad*
*P.O.Box 1436, Mashhad, Iran*

[2] *School of Physics, Institute for Research in Fundamental Sciences (IPM)*
*P.O.Box 19395-5531, Tehran, Iran*

*mnajafizadeh@ipm.ir*

### Appendix A: Schrödinger equation

Let us consider the Klein–Gordon equation for a complex scalar field $\phi$ of mass $m$ in a $(d+1)$-dimensional Minkowski spacetime, with the mostly plus signature for the metric, including the speed of light $c$ and the reduced Planck constant $\hbar$

$$\left(-\frac{1}{c^2}\,\partial_t^2\,+\,\partial^i\partial_i\,-\,\mu^2\right)\phi=0\,, \tag{1}$$

where $\mu := mc/\hbar$. Using a field redefinition

$$\phi=\frac{1}{\sqrt{\mu}}\,e^{-i\mu ct}\,\psi\,, \tag{2}$$

the Klein-Gordon equation (1) reduces to

$$\left(-\frac{1}{c^2}\,\partial_t^2\,+\,\frac{2im}{\hbar}\,\partial_t\,+\,\partial^i\partial_i\right)\psi=0\,. \tag{3}$$

By applying the Galilei or nonrelativistic limit ($c\to\infty$) to the latter, and multiplying by $\hbar^2/2m$, we will arrive at the Schrödinger equation

$$\left(i\hbar\,\partial_t\,+\,\frac{\hbar^2}{2m}\,\partial^i\partial_i\right)\psi=0\,. \tag{4}$$

This equation can be obtain from the Schrödinger action

$$S=\int dt\,d^dx\;\psi^\star\left(i\hbar\,\partial_t\,+\,\frac{\hbar^2}{2m}\,\partial^i\partial_i\right)\psi\,. \tag{5}$$

We note that the length dimension of a scalar field is $[\phi]_{L}=(1-d)/2$. Therefore, following (2), the length dimension for the Schrödinger field $\psi$ reads $[\psi]_{L}=-\,d/2$, and as a result the action (5) possesses the expected dimension of $\hbar$.
